# Acupuncture as Treatment for Female Infertility: A Systematic Review and Meta-Analysis of Randomized Controlled Trials

**DOI:** 10.1155/2022/3595033

**Published:** 2022-02-16

**Authors:** Kewei Quan, Chuyi Yu, Xiaohui Wen, Qiuping Lin, Naiping Wang, Hongxia Ma

**Affiliations:** ^1^Dongguan Hospital of Guangzhou University of Chinese Medicine, Dongguan 523127, Guangdong, China; ^2^Guangzhou University of Chinese Medicine, Guangzhou 510405, Guangdong, China; ^3^Department of Traditional Chinese Medicine, The First Affiliated Hospital of Guangzhou Medical University, Guangzhou 510120, Guangdong, China

## Abstract

**Background:**

The effects of acupuncture on female infertility remain controversial. Also, the variation in the participant, interventions, outcomes studied, and trial design may relate to the efficacy of adjuvant acupuncture. The aim of the study is to systematically evaluate the efficacy and safety of acupuncture for female with infertility and hopefully provide reliable guidance for clinicians and patients.

**Methods:**

We searched digital databases for relevant studies, including EMBASE, PubMed, Cochrane Library, and Web of Science, and the Cochrane Library up to April 2021, for randomized controlled trials (RCTs) evaluating the effects of acupuncture on women undergoing IVF and other treatment. We included studies with intervention groups using acupuncture and control groups consisting of no acupuncture or sham (placebo) acupuncture. Primary outcomes were clinical pregnancy rate (CPR) and live birth rate (LBR). Meta-regression and subgroup analysis were conducted on the basis of ten prespecified covariates to investigate the variances of the effects of adjuvant acupuncture on pregnancy rates and the sources of heterogeneity. *Results:* Twenty-seven studies with 7676 participants were included. The results showed that the intervention group contributes more in outcomes including live birth rate (RR = 1.34; 95% CI (1.07, 1.67); *P* < 0.05), clinical pregnancy rate (RR = 1.43; 95% CI (1.21, 1.69); *P* < 0.05), biochemical pregnancy rate (RR = 1.42; 95% CI (1.05, 1.91); *P* < 0.05), ongoing pregnancy rate (RR = 1.25; 95% CI (0.88, 1.79); *P* < 0.05), adverse events (RR = 1.65; 95% CI (1.15, 2.36); *P* < 0.05), and implantation rate (MD = 1.19; 95% CI (1.07, 1.33); *P* < 0.05) when compared with the control group, and the difference is statistically significant. In terms of the number of oocytes retrieved, good-quality embryo rate, miscarriages, and ectopic pregnancy rate, the difference between the acupuncture group and the control group was not statistically significant. *Conclusions:* Our analysis finds a benefit of acupuncture for outcomes in women with infertility, and the number of acupuncture treatments is a potential influential factor. Given the poor reporting and methodological flaws of existing studies, studies with larger scales and better methodologies are needed to verify these findings. More double-blind RCTs equipped with high quality and large samples are expected for the improvement of the level of evidence.

## 1. Introduction

Infertility is explicitly defined as a failure to become pregnant within 12 months of having regular, unprotected, heterosexual intercourse [1]; it affects approximately 48.5 million couples worldwide [2]. Complementary therapies are widely used by patients with infertility. Acupuncture as a nonpharmacological therapy for women with infertility [3] was first reported in 1988 [1], showing effects similar to those of auricular acupuncture and drug-based therapy for achieving pregnancy, increasing research interest in this method [2–4]. The first systematic review on this subject was published in December 2002 [5] and showed no definitive findings; however, the authors speculated the involvement of the hypothalamic-pituitary-ovarian axis and peripheral uterine stimulation, both of which require further research. Prospective randomized controlled studies are essential to evaluate the effectiveness of acupuncture as a treatment for female infertility. Previously, Paulus et al. conducted a randomized trial (RCT), showing that acupuncture, compared with control treatment involving standard care, doubled the odds of becoming pregnant [6]. Acupuncture may improve pregnancy rates and reduce the levels of stress, anxiety, and depression [7–10]. However, systematic reviews have produced conflicting findings [11–17], likely due to patient and method heterogeneity or small sample sizes; finally, some studies lacked a placebo control group, which is essential to distinguish the impact of an intervention from that of other factors [18]. To better illustrate the efficacy of acupuncture in infertility, we expanded the criteria included in the literature to include not only in vitro fertilization (IVF) but also acupuncture plus drug-assisted pregnancy. In addition, in the subgroup analysis, we included the availability of placebo as a grouping criterion, which has not been attempted in other systematic reviews. Herein, we aimed to conduct a systematic review and meta-analysis of RCTs, including subgroup analyses and meta-regressions, to examine the impact of acupuncture on female infertility.

## 2. Methods

We followed the Preferred Reporting Items for Systematic Reviews and Meta-analysis statement guidelines [19] and formulated a study protocol, which included study objectives, search strategies, inclusion and exclusion criteria, outcome measures, and methods of statistical analysis, before the study was conducted. For this review, data were extracted from the selected literature and analyzed; however, the study was not registered. In this report, we selected RCTs on acupuncture for infertility published in the English language.

### 2.1. Search Strategy

Without any restrictions on languages, categories, or publication types, we retrieved articles from the following databases from their inception to April 2021: PubMed, EMBASE, Web of Science, and the Cochrane Library. However, only studies published in English were included in this review. We employed Medical Subject Heading terms and relevant keywords for the search. The retrieval formula was as follows: (Title/Abstract): female infertility/sterility, assisted reproduction, embryo transfer, in vitro fertilization, polycystic ovary syndrome, acupuncture, pharmacopuncture, electroacupuncture, and needle; we also searched for previous systematic reviews on this topic and reviewed their reference lists [20–23]. In addition, we searched Google Scholar for book publications relevant to infertility and acupuncture and then checked the reference lists for relevant articles; the search strategy was developed after consultation with an experienced medical research professor.

### 2.2. Eligibility Criteria

RCTs comparing the effects of acupuncture with those of sham acupuncture or no acupuncture in adult patients treated for infertility were included. We excluded controlled trials, cohort studies (C), case series, and case studies (Case). Studies were categorized according to the type of control group: acupuncture vs. sham acupuncture and acupuncture vs. no intervention; other trials were excluded, such as acupuncture vs. some medication, real acupuncture with Chinese herbology vs. sham, acupuncture with Chinese herb, and acupuncture with medication vs. medication alone.

### 2.3. Data Extraction and Outcomes of Interest

Two reviewers (Kewei Quan and Chuyi Yu) independently extracted and analyzed eligible study data. Any discrepancies were resolved by consulting a senior author (Hongxia Ma). We used a standardized data extraction form to collect the following data: first author last name, year of publication, country of study, case and control group sizes, mean age of participants, participant's BMI, and acupuncture type; as well as effect size measures (odds ratios (OR) with 95% confidence intervals (CI) were recorded. The study authors were contacted for clarifications, as needed.

Primary outcomes were the rates of biochemical pregnancy, clinical pregnancy (presence of at least one gestational sac or fetal heartbeat, confirmed by transvaginal ultrasound), ongoing pregnancy (pregnancy beyond 12 weeks of gestation, as confirmed by fetal heart activity on ultrasound), and live births. Secondary outcomes were the rates of adverse events, implantation, miscarriage, ectopic pregnancy, and the number of good-quality embryos; in addition, endometrial thickness and the number of retrieved oocytes were evaluated.

### 2.4. Quality Assessment and Statistical Analysis

We assessed each study included in the systematic review for the risk of bias using the Cochrane Collaboration assessment tool [24], which included seven items related to random sequence generation and allocation concealment, blinding of participants and personnel, outcome assessment, incomplete outcome data, selective outcome reporting, and other sources of bias. The studies were rated in each domain as at low, high, or unclear risk of bias; each study was rated on a scale of 1–7 points, where a score of 5–7 points indicated a high-quality study.

All analyses were performed using Review Manager 5.6 (Cochrane Collaboration, Oxford, UK) and STATA 12.0 (StataCorp, College Station, TX, USA). We used the weighted mean difference to analyze continuous variables, and the OR was used as the summary statistic for dichotomous variables. For studies that published their findings as mean values with ranges, standard deviations were calculated using statistical algorithms. Heterogeneity among cases was evaluated by the chi-square test with significance set at *P* values of <0.10; if heterogeneity among studies was high, we used the random-effects model; otherwise, we used the fixed-effects model.

Subgroup analyses were performed according to the type of control group (sham acupuncture or blank control). As there were >10 trials included in the analysis, sensitivity analyses were used for high quality; funnel plots were used to assess potential publication bias.

## 3. Results

**Figure 1:**
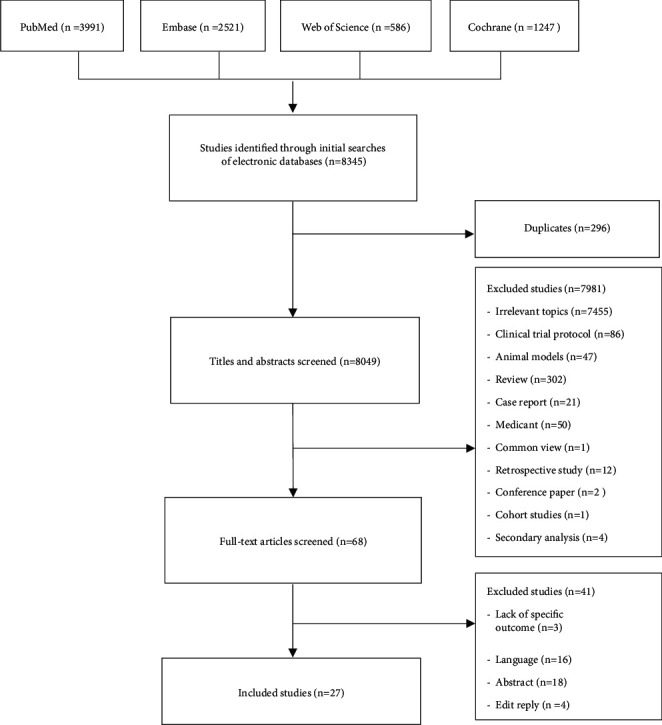
Process of searching and screening studies.

A total of 25 full-text articles and 2 conference reports met the eligibility criteria and were included in the analysis (Figure 1). First, study titles and abstracts were screened, and then full texts of eligible studies were retrieved from databases for further evaluation. The preliminary browsing of database produced 8345 articles, including 296 duplicates, which were removed. In the remaining literature, 7981 cases were excluded based on information included in their titles and abstracts. Some studies had control groups that received pharmaceutical or herbal medicine or oral contraceptives, which may interfere with the effects of acupuncture, so we excluded these studies. In the literature review, we searched a relevant literature in a variety of languages, but to ensure consistency, we included only studies published in the English language. We included RCTs that compared the impact of true acupuncture with that of sham acupuncture or no intervention in women with infertility undergoing ovulation induction, in vitro fertilization (IVF), or intracytoplasmic sperm injection. To evaluate the impact of the level at which acupuncture was administered, we included studies that reported acupuncture placement, specifically, the meridian point with inert point or nonmeridian point. Nonrandomized trials, retrospective comparative studies, conference abstracts, and observational studies were excluded. After full-text screening, an additional 41 studies were removed. In total, 27 published studies were included in the meta-analysis (Table 1).

### 3.1. Study Design

Two authors (Kewei Quan and Chuyi Yu) independently selected and reviewed all studies; any disagreements were resolved by discussion.

The characteristics of the included studies are summarized in Table 1. A total of 27 RCTs evaluated a total of 7676 cases (4375 cases and 3301 controls); these studies included 25 full-text articles [6, 7, 9, 10, 25–45] and 2 conference abstracts [46, 47]. Twenty-five studies comprehensively examined the causes of infertility, including male-related and tubal factors, endometriosis, and other factors, including PCOS and unclear causes [6, 7, 9, 10, 25–32, 34–38, 40–47]; two studies reported PCOS as the cause of infertility [33, 39]. The mean age of participants was reported in 26 studies [6, 14–32, 34–39] and ranged from 28 to 38 years. Baseline characteristics of the groups were comparable in each study.

### 3.2. Interventions

Five trials compared the effectiveness of manual and noninsertive manual acupuncture [25, 26, 28, 29, 42]. Two trials used electroacupuncture [33, 39], while three used auricular acupressure [7, 32, 37]. One trial used MA + moxibustion [35]. Nine studies compared real acupuncture vs. sham acupuncture [26,28,29,37,39,41–43,47], twelve used blank groups as controls [6, 9, 10, 27, 30, 33, 34, 36, 41, 44–46], and five observed the impact of acupuncture at different stages before and after transplantation [6, 10, 30, 31, 44]. Three forms of placebo acupuncture were used. First was the method used by Wu et al., which involved a superficial insertion in the shoulder and upper arm without manual or electrical stimulation [39]. The second type involved blunt acupuncture with the blunt tip of the needle [25, 26, 28, 29, 42], which was not fixed into the copper handle and was retractable. When the needle was pushed forward against the skin, it slid into the handle, and the entire needle appeared shortened. The third type involved acupuncture at acupoints and meridians unrelated to fertility [37, 47] and not necessarily on the shoulder.

### 3.3. Study Quality

**Figure 2:**
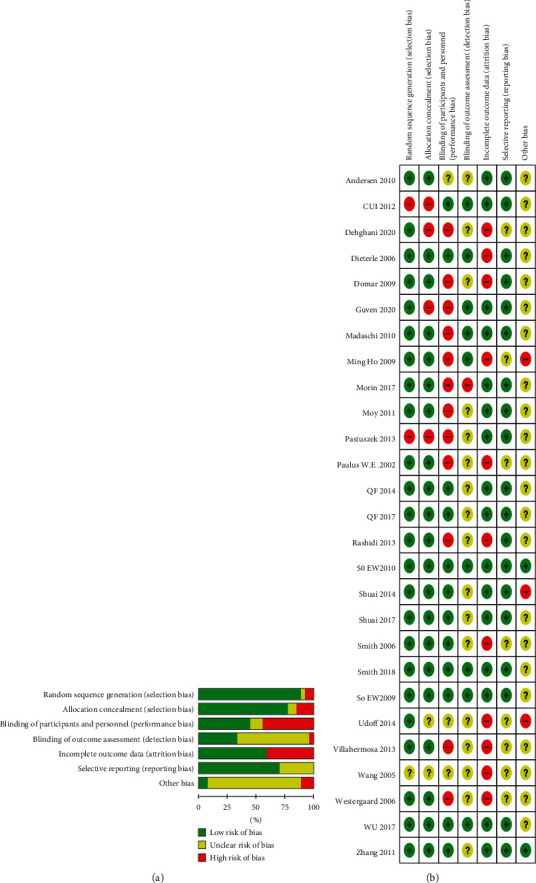
Risk of bias summary and risk of bias graph.

Majority of the trials included in this review were of high quality, with two exceptions [46, 47] that were conference abstracts lacking information on randomization procedures, among others. The included studies scored 7 points (Figure 2). Eleven studies [7, 26, 28, 30, 31, 37–40, 42,43] presented most of the required information and were judged as of high quality. One study [36] failed to adequately describe randomization and blinding procedures; another study [35] used moxibustion in the treatment group without providing an adequate control; thus, both studies were considered of low quality.

### 3.4. Primary Outcomes

**Figure 3:**
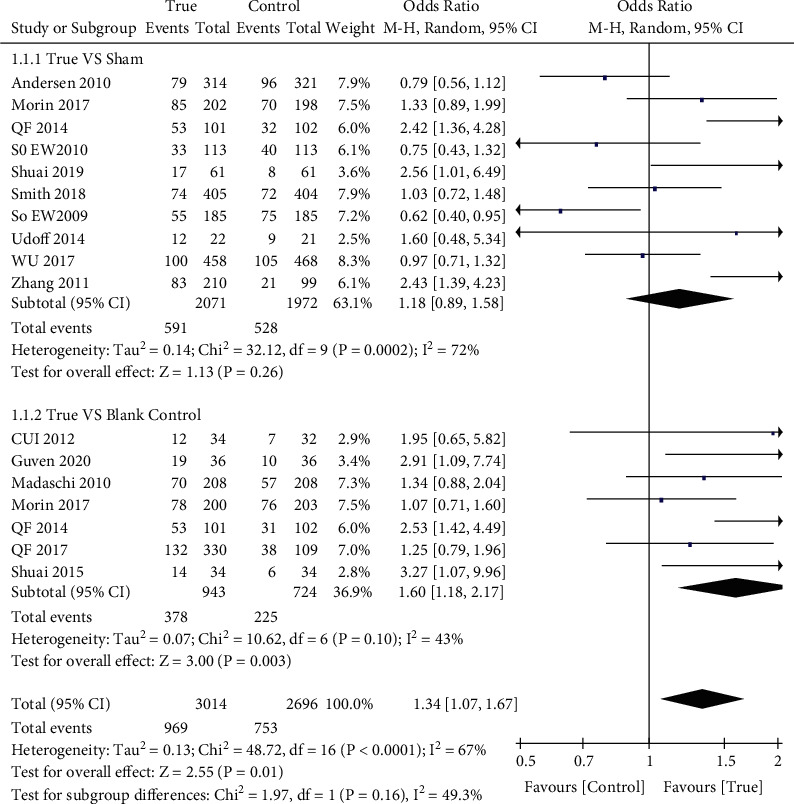
Forest plot of the live birth rate (all types of interventions).

**Figure 4:**
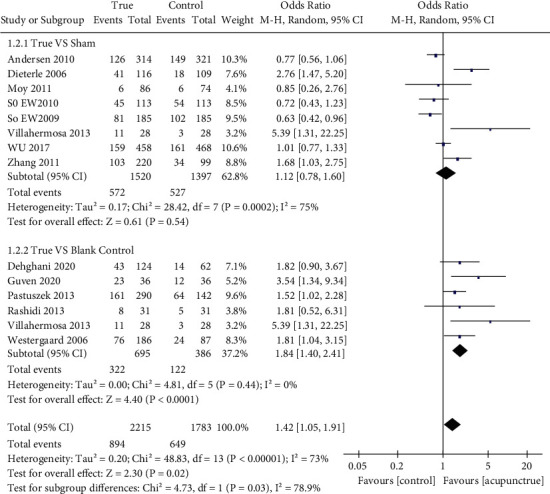
Forest plot of the biochemical pregnancy rate (all types of interventions).

**Figure 5:**
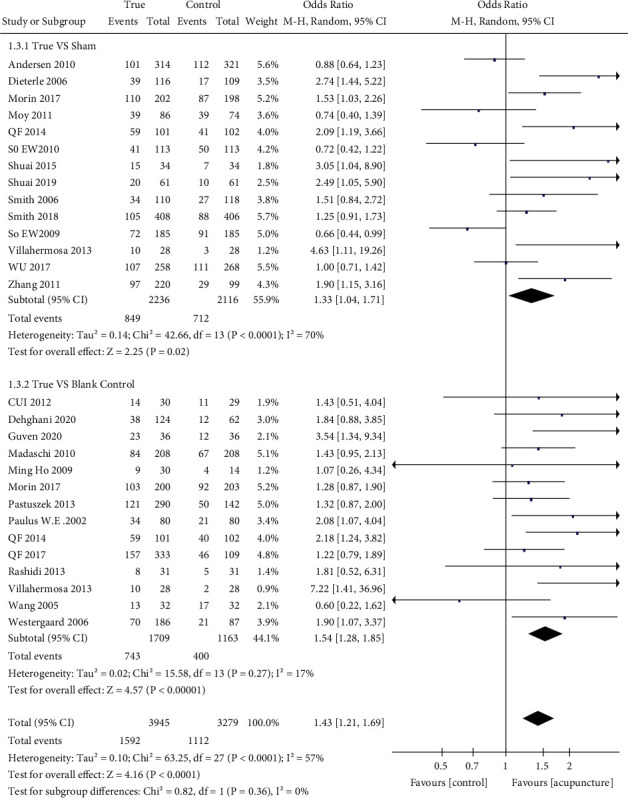
Forest plot of the clinical pregnancy rate (all types of interventions).

We summarized four indicators as primary outcomes (Table 2). Fifteen studies [26, 28–31, 33, 37–43, 45, 47] examined live birth rate (LBR) in patients (*n* = 5710) assigned true acupuncture or sham acupuncture; the LBR in the acupuncture group was higher than that in the control group (32.1% and 27.9%; OR: 1.34; 95% CI: 1.07–1.67; *P*=0.01) (Figure 3). Biochemical pregnancy rates were available in 13 studies [7, 10, 26, 28, 29, 31, 32, 34–36, 39, 44, 45], and there were significant differences in these rates between the groups (true acupuncture group: 40.4% and control group: 36.4%; OR: 1.42; 95% CI: 1.05–1.91; *P*=0.02) (Figure 4). All included studies [6, 7, 9, 10, 25–47] examined clinical pregnancy rates; however, two studies [27, 47] failed to report them. Consequently, 25 studies (*n* = 7224) were included; the rates of pregnancy were different between the true and control groups (40.4% and 33.9%; OR: 1.43; 95% CI: 1.21–1.69; *P* < 0.0001) (Figure 5).

**Figure 6:**
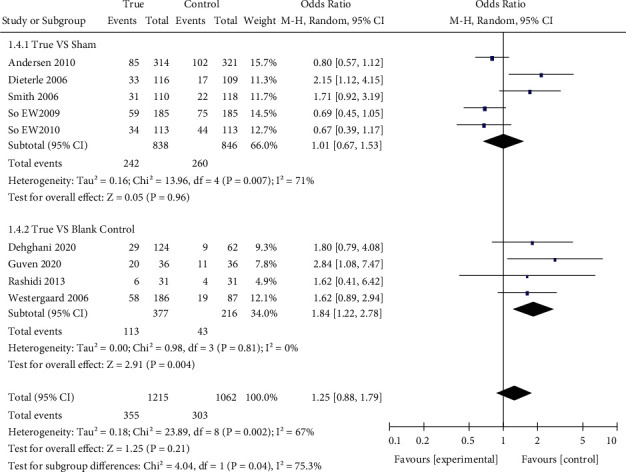
Forest plot of the ongoing pregnancy rate (all types of interventions).

Nine studies [7, 10, 25, 26, 28, 29, 34, 44, 45] reported ongoing pregnancy rates (*n* = 2277), which were similar in both groups (29.2% and 28.5%; OR: 1.25; 95% CI: 0.88–1.79; *P*=0.21) (Figure 6).

### 3.5. Secondary Outcomes

**Figure 7:**
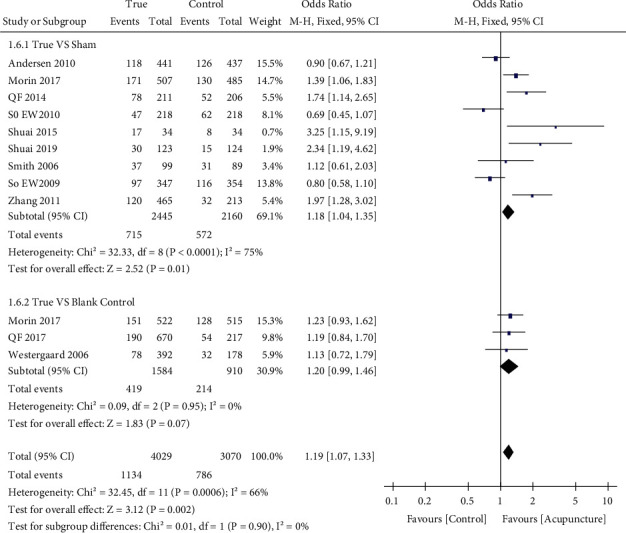
Forest plot of the implantation rate (all types of interventions).

Eleven studies [10, 25, 26, 28, 29, 31, 37, 38, 40, 41, 43] reported implantation rates (*n* = 7099); the acupuncture group rates were higher than the control group rates (28.1% and 25.6%; OR: 1.19; 95% CI: 1.07–1.33; *P*=0.002) (Figure 7). Four studies [26, 28, 39, 42] assessed adverse events (*n* = 2204) and reported slightly higher adverse event occurrences such as local pain, bleeding, bruising, and pruritus, in the true acupuncture group than in the control group (53.8% and 44.7%; OR: 1.65; 95% CI: 1.15–2.36; *P*=0.006), with moderate among-study heterogeneity (*χ*^2^ = 9.65, df = 3, *P*=0.02; I^2^ = 69%). However, there was no difference between the groups in good-quality embryo rates [29], number of retrieved oocytes [9, 25, 29, 30, 32–35, 37, 40, 41, 43, 45], miscarriage incidence [10, 26, 28, 30, 31, 33, 34, 36, 39, 42], or ectopic pregnancy rates [26, 36, 39].

### 3.6. Subgroup Analyses

There was no difference in live birth rates between the true and sham acupuncture groups (*n* = 4043) [26, 28, 29, 31, 37, 39, 41–43, 47] (OR: 1.18; 95% CI: 0.89–1.58; *P*=0.26). However, there was a significant difference in this outcome between the true acupuncture and blank control groups [30, 33, 37, 38, 40, 41, 45] (*n* = 1667) (OR: 1.60; 95% CI: 1.18–2.17; *P*=0.003). However, one study [37] used both sham needles and blank controls and was included twice in the analysis; excluding this article did not affect the overall results.

In addition, there was no significant difference in biochemical pregnancy rates between the sham and true acupuncture needle groups [7, 26, 28, 29, 31, 32, 35, 39] (OR: 1.12; 95% CI: 0.78–1.60; *P*=0.54). However, studies that contained a blank control group [10, 34–36, 44, 45] (*n* = 1081) revealed a higher rate of biochemical pregnancy in the true acupuncture group than in the blank control group (46.3% vs. 31.6%; OR: 1.84; 95% CI: 1.40–2.41; *P* < 0.0001).

There was a small difference in clinical pregnancy rates between the sham and true groups [7, 25, 26, 28, 29, 31, 32, 35, 37–39, 41–43] (38.0% vs. 33.6%; OR: 1.33; 95% CI: 1.04–1.77; *P*=0.02). However, the true acupuncture group had a higher rate of clinical pregnancy than did the blank group [9, 10, 30, 33–38, 40, 41, 44–46] (*n* = 2872) (43.5% vs. 34.4%; OR: 1.54; 95% CI; 1.28–1.85; *P* < 0.00001).

The ongoing pregnancy rates were similar in the sham and true acupuncture groups [7, 25, 26, 28, 29] (*n* = 1684) (28.9% vs. 30.7%; OR: 1.01; 95% CI: 0.67–1.53; *P*=0.96). However, there were significant differences in the ongoing pregnancy rates in four studies [10, 34, 44, 45] (30.0% vs. 19.9%; OR: 1.84; 95% CI: 1.22–2.78; *P*=0.004).

### 3.7. Sensitivity Analysis and Publication Bias

Sensitivity analyses included 11 RCTs [7, 26, 28, 30, 31, 37–40, 42, 43] that scored ≥5 points on the Cochrane Collaboration assessment tool, except for one study [31] that performed group assignment before and after transplantation, which was different from the method used in the other studies (Table 3). Only outcomes reported in three or more studies were included in the sensitivity analysis. Sensitivity analyses did not affect any of the estimates, except for the adverse event rate, which was higher in the true group than in the control group.

**Figure 8:**
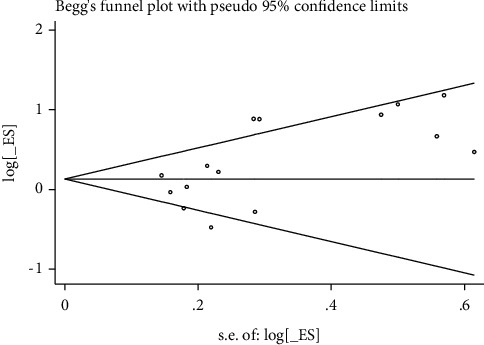
Live birth rate.

**Figure 9:**
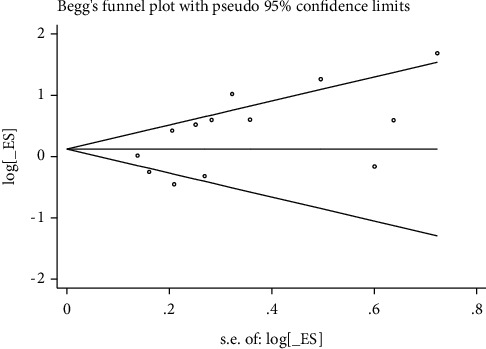
Biomechanical pregnancy rate.

**Figure 10:**
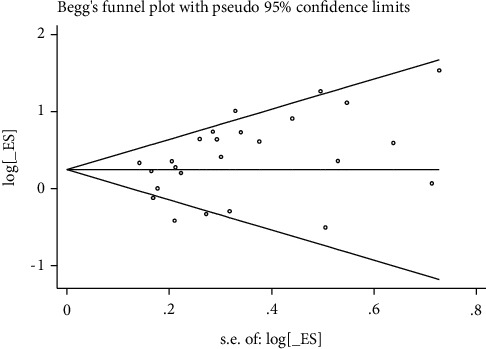
Clinical pregnancy rate.

**Figure 11:**
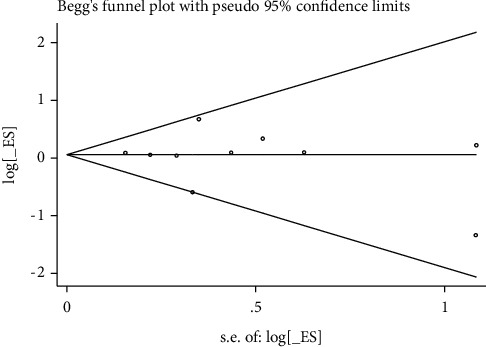
Miscarriage rate.

These Egger tests revealed some publication bias in studies reporting the rates of live birth, biochemical pregnancy, clinical pregnancy, and miscarriage (Figures 8–11).

## 4. Discussion

In this systematic review, we identified 27 RCTs (*n* = 7676, including 4375 and 3301 cases and controls, respectively) that investigated the impact of acupuncture on reproductive outcomes. Regarding the main observational indicators, we included more relatively large studies, including 15 studies evaluating live birth rates, 25 evaluating clinical pregnancy rates, 13 evaluating biochemical pregnancy rates, and 9 evaluating ongoing pregnancy rates. The number of studies included in this review was higher than that in similar previously published meta-analyses. The results showed that acupuncture, compared with control treatment, improved the live birth rate, biochemical pregnancy rate, clinical pregnancy rate, and implant rate in infertile patients. However, acupuncture did not show beneficial outcomes in other pregnancy-related factors such as ongoing pregnancy rate, oocytes retrieved, good-quality embryo rate, miscarriages, and ectopic pregnancy rate. We also found that the incidence of adverse events in the acupuncture group was significantly higher than that in the control group. We found clear advantages of acupuncture over blank control conditions in terms of the live birth rate, biochemical pregnancy rate, ongoing pregnancy rate, and clinical pregnancy rate. However, these effects were similar between the true and sham acupuncture groups, and the rate of adverse events was lower in the sham group than in the true acupuncture group.

Live birth rates are considered key outcomes in studies in infertility. The present findings suggest that true acupuncture is unlikely to improve live birth rates compared to those associated with sham acupuncture; however, live birth rates were higher in the acupuncture group than in the blank control group. These results were unexpected, as sham acupuncture was used on acupoints unrelated to reproductive function or with nonirritating needles or patches placed on the relevant acupoints but without giving qi stimulation, as required by traditional Chinese medicine (TCM) theory [7, 25, 26, 28, 29, 32, 35, 37–39, 42, 43, 47]. Nevertheless, the effects were comparable in both conditions. Blunt acupuncture may trigger a psychological placebo effect similar to that observed in a pharmacologically negative placebo group. Sham acupuncture that is not blunt may not act as a placebo and may not change the levels of neurotransmitters; however, it does cause microinjury and increases local blood flow. The present findings suggest that the physical placebo may be as safe and as effective as infertility treatment as true acupuncture.

It should be noted that live birth rates depend on ovarian function and are affected by several parameters, including metabolic abnormalities, uterine condition, pelvic surgery history, and sperm quality. In the present study, the effects of true and sham acupuncture on live birth rates were similar; these findings may be accounted for by the placebo effect, or the stress relief associated with acupuncture [48].

Six studies [6, 10, 30, 31, 36, 44] compared either the timing of acupuncture treatment, some groups were treated with acupuncture before transplantation, some with acupuncture after transplantation, and some with acupuncture before and after transplantation, or compared the levels of the intensity and frequency of acupuncture [40]. The benefit of acupuncture was greater than that of no intervention for clinical pregnancy rate, but the effects on live birth rates were negligible. In addition, a study [40] concluded that the clinical pregnancy rate, implantation rate, and live birth rate of the TEAS-2/100 Hz group were significantly higher than those of the other groups. However, larger studies are needed to confirm that using a frequency of 2/100 Hz electroacupuncture may improve IVF outcomes.

In the present study, there was no impact of true acupuncture on biochemical or ongoing pregnancy rates. However, clinical pregnancy rates were higher in the true group than in the sham or nonintervention groups. In addition, implantation rates were higher in the true group than in the sham group and similar to those in the blank control group; this finding may be accounted for by the small sample size. Meanwhile, the rates of adverse events were higher in the true group than in the sham group, which may be due to the true acupuncture requirement to target many points that are deep within the tissue, and which receive relatively high levels of electric stimulation. These requirements contrast with those of sham acupuncture, which involve fewer and more superficially located stimulation points, reducing the risk of adverse reactions. Nevertheless, the present findings suggest that sham and true acupuncture are comparably safe and effective for some outcomes. There was no between-group difference in the rates of implantation or miscarriage, or a number of oocytes retrieved or that of good-quality embryos.

To evaluate the impact of literature quality on this review, we performed a sensitivity analysis on the 10 studies we considered were of the highest quality. This analysis revealed that acupuncture does not affect fertility outcomes. However, although this study included several RCTs, those of high quality were limited. The selection of infertility patients, intervention methods, and acupuncture points led to limitations in the study results. This also highlights the importance of high-quality literature for meta-analysis.

The studies we selected were clinical randomized controlled studies with appropriate research methods. Almost all of the studies were designed in detail with the exception of two conference papers [46, 47]; therefore, we extracted more relevant observation indicators. One of the main limitations of our meta-analysis is that the target intervention, i.e., acupuncture, varied among patients and included manual acupuncture [6, 7, 9, 10, 25–30, 32, 34–36, 39, 41, 42, 44–47], electroacupuncture [33], aural acupuncture [37], transcutaneous electroacupuncture [38, 40], and laser acupuncture [41]. Each type is considered as acupuncture according to the TCM theory. Furthermore, there are great differences in the selection of the acupuncture points. In terms of TCM theory, different meridians and acupoints have different functions. Another limitation of this review is that we included studies reporting live birth rates after IVF and ovulation induction treatments; this may have introduced bias. Furthermore, there were differences in the choice of treatment and observation groups among studies. For example, Wu et al. divided the participants into 4 groups: true acupuncture plus clomiphene, control acupuncture plus clomiphene, true acupuncture plus placebo, and control acupuncture plus placebo [39]. Some trials divided the participants into just two groups: the real needle group and the placebo-needle group [7, 25, 26, 28, 29, 32, 35, 37, 42, 47]. Some divided the participants into a real needle group and a no-acupuncture group [6, 9, 27, 30, 33, 34, 45, 46]. Finally, other studies [6, 10, 30, 31, 36, 44] divided the participants according to the timing of acupuncture treatment.

In addition, although all included studies involved acupuncture, the details of the procedures, including stimulus type or intensity, were not always reported, shifting the focus to true vs. false vs. no needle comparisons. Furthermore, the selection of acupuncture points, the stimulus intensity, stimulation technique, etc., were not provided in detail. In TCM, the selection of acupoints should be individualized based on the presentation of the disease condition. In these RCTs, this principle was not used as the same acupuncture program had to be used for all patients in a group. This could have affected the results of their studies. In addition, all the RCTs had no follow-up data; therefore, the long-term effects of the acupuncture treatment were not reported. Last, the sample size in this study was small, and RCTs with larger samples and more detailed grouping are warranted to support this evidence.

Nevertheless, the present meta-analysis presents the most up-to-date findings in this field. This study involved rigorous eligibility criteria and comprehensive literature search; the dataset was large, and the analytical methods used were valid, yielding robust and reliable findings.

In summary, this review provides moderate evidence of the benefits of acupuncture for infertile women; this will enable medical researchers to consider using acupuncture to help infertile women conceive in future clinical practice. The present findings suggest that true acupuncture does not affect female fertility outcomes. However, the blunt needle use may be superior to true acupuncture at improving live birth rates. Evidence for the use of blunt acupuncture to treat infertility is insufficient.

## Figures and Tables

**Table 1:** Characteristics of the included studies.

	Study	Country	Participants	Age (year)	BMI	Acupuncture type	Acupuncture session	Outcomes
			Acu	Control	Acu	Control	Acu	Control	Acu	Control	Acu	Control	
1	Wu 2017	China	Active Acu + clomiphene: 250	Control Acu + Clomiphene: 250	Active Acu + clomiphene: 28.2 (3.4)	Control Acu + Clomiphene: 27.8 (3.4)	Active Acu + clomiphene: 23.8 (4.2)	Control Acu + Clomiphene: 24.4 (3.9)	MA + EA	NMA + NEA	Real acu: located in abdominal muscles and leg muscles, and in the hands and head	Sham acu: in each shoulder and upper arm at nonacupuncture points	LBR, RO, conception, pregnancy, and multiple pregnancy, AE
			Active Acu + placebo: 250	Control Acu + Placebo: 250	Active Acu + placebo: 27.8 (3.2)	Control Acu + placebo: 28.0 (3.3)	Active Acu + placebo: 24.2 (4.4)	Control Acu + Placebo: 24.6 (4.5)					

2	SO EW2010	China HK	113	113	35 (3.7)	35 (2.96)	: 21.6 (2.3)	21.9 (2.6)	MA	MA (Streitberger placebo-needle)	SAME	SAME	OPR CPR, OPR, LBR, IR, MR

3	Smith 2018	Australia and New Zealand	424	424	35.4 (4.3)	35.5 (4.3)	25.8 (5.5)	26.0 (5.8)	MA	Noninsertive acupuncture	Real acu: be beneficial to the uterus and ovaries	Sham acu: away from known acupuncture points and with no known function	LBR, CPR, AE

4	QF 2017	China	TEAS-2 Hz group: 108	Control group: 109	TEAS-2 Hz group: 31.22 (5.92)	29.81 (6.17)	TEAS-2 Hz group: 22.97 (6.59)	21.53 (6.28)	TEAS-2 Hz group	NO TEAS treatment	SP10, SP8, LR3, ST36, EX-CA1,CY4, PC6,CY12	NO TEAS treatment	CPR, LBR, IR, NMO, NFO, GQE
			TEAS-100 Hz group: 111		TEAS-100 Hz group: 21.77 (5.98)		TEAS-100 Hz group: 21.77 (5.98)		TEAS-100 Hz group				
			TEAS-2/100 Hz group: 114		TEAS-2/100 Hz group: 31.16 (6.09)		TEAS-2/100 Hz group: 23.14 (6.55)		TEAS-2/100 Hz group: 114				

5	Villahermosa 2013	Brazil	28	28	36.0 (2.7)	36.2 (2.2)	NR	NR	MA + MB	MA	Real acu: manual manipulation AND moxibustion (the principles of traditional Chinese medicine and the classic point localization, including depth of insertion)	Sham acu: nonmeridian and shallow stimulus AND no moxibustion (performed in the arm and thigh)	BPR, CPR

6	Udoff 2014	USA	31	29	: 32.4	33.2	NR	NR	MA	MA	Real acu: meridia and manual manipulation	Sham acu: nonmeridian and shallow stimulus	CPR, DR

7	Moy 2011	USA	86	74	33.3 (0.307)	33.16 (0.334)	24.77 (1.051)	24.05 (0.582)	MA + AA	MA + AA	Real acu: in qi lines (CV6, SP8, LR3, ST29, GV20, HT7)+AA55, AA50, AA58, AA22	Sham acu: in non-qi lines (near the acupoints above) + knee, heel, allergic area, mouth	CPR, McG-SPDt

8	Andersen 2010	Denmark	314	321	31 (?)	31.16 (?)	22.5 (?)	22.5 (?)	MA	MA (Streitberger placebo-needle)	SAME	SAME	P-HCG, CPR, OPR, LBR

9	Dieterle 2006	Germany	116	109	35.1 (3.8)	34.7 (4.0)	24.5 (5.1)	24.1 (4.7)	MA + AA	MA + AA	The true acupuncture treatment was designed to influence fertility closely	The placebo acupuncture treatment was	BPR, CPR, OPR
												designed not to influence fertility	

10	Smith 2006	Australia	110	118	35.9 (4.7)	36.1 (4.8)	25.4 (4.2)	26.0 (5.6)	MA	MA (Streitberger placebo-needle)	Acupuncture was administered	These were located close to but not on the real acupuncture points. Because the tip	CPR, OPR, implantation, AE, HS
											with point selection based on the TCM diagnosis.	of the needle is blunted, skin penetration did not occur	

11	So EW 2009	China HK	185	185	36 (3.704)	36 (2.963)	21.6 (2.1)	21.7 (2.7)	MA	MA (Streitberger placebo-needle)	SAME	SAME	OPR, CPR, OPR, LBR, IR, MR, EPR

12	Domar 2009	America	78	68	36.1 (?)	36.1 (?)	NR	NR	MA	No treatment	The same 22-needle points were chosen for their sedative effect as well as to increase uterine blood flow.	No treatment	CPR, anxiety, optimism.

13	Guven 2020	Turkey	36	36	30.3 (3.4)	31.5 (4)	24.4 (3.0)	23.3 (1.9)	MA	No treatment	H7, LI4, GV20, ear AA55, CV3, CV4, CV6, LIV3, ST30, and SP8, bilateral LI4, SP6, SP9, ST36	No treatment	*β*-HCG level, CPR, OPR,LBR, anxiety level

14	Dehghani 2020	Iran	ACU1: 62	62	ACU1: 32.1 (5.9)	31.5 (5.4)	ACU1: 25.1 (3.3)	26.3 (3.9)	ACU1 : acupuncture 25 min before ET	ET without acupuncture	HT7,PC6, CV6, GV20, SP6,CV4	No acutreatment	BPR. CPR. OPR
			ACU2: 62		ACU2: 32.9 (4.8)		ACU2: 25.2 (3.8)		ACU2: acupuncture 25 min before and after ET				

15	Paulus W.E.2002	Germany	80	80	32.8 (4.1)	32.1 (3.9)	NR	NR	MA 25 min before and after ET	ET without acupuncture	Before: PC6, SP8, LR3, GV20, ST29. After: ST36, SP6, SP10, LI4	No acutreatment	CPR

16	Ming Ho 2009	Taiwan	30	14	35.5 (4.5)	34.0 (5.2)	NR	NR	EA	No acupuncture	LR3, SP6, ST28, EX-CA1, CV6, CV4	No acutreatment	CPR, PI

17	Madaschi 2010	Brazil	208	208	35.3 (4.7)	34.6 (4.6)	22.4 (3.8)	22.4 (2.9)	MA 25 min before and after ET	No acupuncture	Before ET PC6, SP8, LR3 GV20, ST29. After ET ST36, SP6, SP10, Li4	No acutreatment	CPR,IR,AR

18	Shuai 2014	China	34	34	29.47 (3.24)	29.65 (2.60)	21.99 (2.71)	22.32 (1.64)	TEAS	Mock TEAS	CV3, CV4. and SP6 and EX-CA1 bilaterally	Same	E Tri-L. ET. IHCs. EV. EVI. SVIP

19	Westergaard 2006	Denmark	ACU 1 : 95	87	ACU 1 : 37 (24–45)	37 (27–45)	ACU 1 : 23 (16–40)	23 (18–32)	ACU 1: acupuncture was given on the day of ET ET	No acupuncture	DU20 ST29, SP8, PC6, and LR3. ST36, SP6, SP10, LI 4	No acutreatment	CPR, OPR
			ACU 2 : 91		ACU 237 (27–45)		ACU 2 : 22 (18–34)		ACU 2: acupuncture was given on the day of ET and duration 2 days after ET		DU20, ST29, SP8, PC6, and LR3. ST36, SP6, SP10, LI 4, and DU20, Ren 3, ST29, SP10, SP6, ST36, and LI 4.		

20	Shuai 2017	China	61	61	31.23 ± 3.78	31.58 ± 3.07	22.01 ± 1.81	22.39 ± 2.87	TEAS	Mock TEAS	SP6, CV3, CV4 and EX-CA1	Same	IR, CPR, LBR

21	CUI 2012	China	34	32	29.3 ± 3.7	29.3 ± 3.45	24.24 ± 4.13	23.96 ± 3.14	EA	No acupuncture	CV4, CV3, SP6, EX-CA 1, KI3	No acutreatment	CPR, LBR, FR, CR, CCR, EMR

22	QF 2014	China	101	102	31.65 (4.30)	30.87 (4.12)	22.08 (3.55)	21.01 (4.25)	AA	AA (auricular acupressure)	AA : AA55, AA22, AA30	Sham AA : triple energizer, stomach, large intestine	CPR.LBR.IR

23	Zhang 2011	China	Single TEAS: 110	Mock TEAS: 99	31.9 (5.3)	31.5 (5.2)	23.2 (3.0)	22.6 (3.5)	Single TEAS treatment: 30 minutes after ET	30 minutes after ET	ST36 and KI3, BL23, RN 4	Same	CPR, IR, LBR
			Double TEAS: 100		32.6 (4.9)		22.6 (3.4)		24 hours before ET and 30 minutes after ET				

24	Rashidi 2013	Iran	31	31	31.03 ± 4.82	32.10 ± 4.68	27.83 ± 4.61	26.10 ± 4.15	MA	No acupuncture	LI4, SP6, LR3, CV4, GV20, ST36	No acutreatment	BPR, CPR, OPR, MR

25	Moring 2017	America	Needle acu: 200	203	NR	NR	NR	NR	Needle acu	No acupuncture	CV6, SP8, LR3, GV20, ST29. After ET : ST36, SP6, SP10, LI4	No acutreatment	BPR, CPR, EPR, MR
			Laser acu: 202						Laser acu				
			Sham laser acu: 198						Sham laser acu				

26	Pastuszek 2013	Poland	Group 1 : 148	Group 3 : 142	32.9 (3.2)	32.7 (3.4)	21.9 (2.4)	22.5 (2.8)	MA during stimulation and on the day of ET	No acupuncture	ST6, HE7 PC6 KI6 or KI3 AA22 AA55 AA58, GV20, EX18, Ren3, Ren4, Ren5, Ren6	No acutreatment	IR, CPR, LBR
			Group 2 : 142		33.2 (3.3)		22.0 (2.5)		MA only on the day of ET;		HE7, PC6, EX1, GV20 AA22, AA58 ST29 or ST30, KI6, Ren3, Ren4, Ren5, Ren6; and after ET (30 min): LI4, EX1, GV20, AA22, ST36 KI3 or KI6, LR2, LR3, Ren15		

27	Wang 2005	America	32	32	37.9	36.7	NR	NR	MA	No acupuncture	CX6, GB8 GB9, ST36 SP10, SP8LIV3 S29, R4, R6. After ET : ST36 SP10 SP9, LIV3, BL23	No acutreatment	CPR, LBR

BMI, body mass index (calculated as weight in kilograms divided by height in meters squared); Acu, acupuncture; MA, manual acupuncture; EA, electric acupuncture; NMA, nonmanual acupuncture; NEA, nonelectric acupuncture; NR, no record; LBR, live birth rate; RO, rates of ovulation; AE, adverse events; OPR, overall pregnancy rate; CPR, clinical pregnancy rate; OPR, ongoing pregnancy rate; LBR, live birth and rate; IR, implantation rate; MR, miscarriage rate; NMO, number of mature oocytes; NFO, normally fertilized oocytes; GQE, good-quality embryos; BPR, biochemistry pregnancy rates; DR, delivery rate; McG-SPD, the McGill survey of pain and discomfort; P-HCG, positive human chorionic gonadotrophin; HS, health status; EPR, ectopic pregnancy rate; *β*-HCG, beta human chorionic gonadotrophin; PI, pulsatility index; AR, abortion rates; *E* Tri-L, endometrial triple-line; ET, endometrial thickness. IHCs, immunohistochemistry score (percentage of immunostained cells ×  intensity of nuclear staining); EV, endometrial volume; EVI, endometrial vascularization index; SVIP, subendometrial vascularization index pattern; FR, fertilization rate; CR, cleavage rate; CCR, cycle cancellation rate; EMR, early miscarriage rate; ET, embryo transfer; TCM, Traditional Chinese Medicine; TEAS, transcutaneous electrical acupoint stimulation; AA, auricular acupressure. Acupuncture session: HE7 (Shenmen); PC6 (Neiguan); SP8 (Diji); SP6 (Sanyingjiao); SP9 (Yinlingquan); SP10 (Xuehai); LR3 (Taichong); LIV3(Taichong); DU20 (Baihui); GV20 (Baihui); ST29 (Guilai); ST36(Zusanli); ST6 (Jiache); ST28 (Shuidao); ST30 (Qichong); LI4 (He gu); RN3 (Zhongi); RN4 (Guanyuan); RN5 (Shimen); RN12 (Zhongwan); RN15 (Jiuwei); CV3(Zhongji); CV4(Guan yuan); CV6 (Qihai); EX1 (Taiyang); EX-CA1 (Zigong); EX18 (Dongming); KI3 (Taixi); KI6 (Zhaohai); BL23(Shenshu); GB8(Shuaigu); GB9(Tianchong); AA22 (Neifenmi); AA55 (Shenmen); AA58 (Zigong); AA30 (Genitals); AA50 (Sympathetic).

**Table 2:** Results of the meta-analysis comparison between true acupuncture and control groups.

Outcomes of interest	Studies, no.	True Acu patients, no.	Control patients, no.	WMD/OR (95% CI)	*P* value	Study heterogeneity			
						*χ* ^2^	*df*	*I* ^2^, (%)	*P* value
Primary outcomes									
Live birth rate	15	3014	2696	1.34 (1.07–1.67)	0.01	48.72	16	67	<0.0001
Biochemical pregnancy rate	13	2215	1783	1.42 (1.05–1.91)	0.02	48.83	13	0	<0.0001
Clinical pregnancy rate	25	3945	3279	1.43 (1.21–1.69)	<0.0001	63.25	27	57	<0.0001
Ongoing pregnancy rate	9	1215	1062	1.25 (0.88–1.79)	0.21	23.89	8	67	0.002

Secondary outcomes									
Implantation rate	11	4029	3070	1.19 (1.07–1.33)	0.002	32.45	11	66	0.0006
Oocytes retrieved	13	1666	1633	0.12 (-0.30–0.53)	0.58	22.29	7	0	0.83
Good-quality embryo rate	1	314	321	0.82 (0.59–1.15)	0.26	—	—	—	—
Miscarriages	10	917	648	1.09 (0.84–1.41)	0.5	9.15	9	2	0.42
Adverse events	4	1099	1105	1.65 (1.15–2.36)	0.006	9.65	3	69	0.02
Ectopic pregnancy rate	3	411	330	1.77 (0.53–5.93)	0.36	0.53	2	0	0.77

**Table 3:** Sensitivity analysis comparison between true acupuncture and control groups.

Outcomes of interest	Studies, no.	True Acu patients, no.	Control patients, no.	WMD/OR (95% CI)	*P* value	Study heterogeneity			
						*χ* ^2^	*df*	*I* ^2^, (%)	*P* value
Primary outcomes									
Live birth rate	9	1895	1684	1.20 (0.90–1.60)	0.21	24.6	8	67	0.002
Biochemical pregnancy rate	4	872	875	1.02 (0.62–1.69)	0.93	15.93	3	81	0.001
Clinical pregnancy rate	10	2026	1811	1.30 (0.98–1.71)	0.07	40.76	11	73	<0.0001
Ongoing pregnancy rate	3	414	407	0.97 (0.49–1.89)	0.92	9.32	2	79	0.009

Secondary outcomes									
Implantation rate	6	1814	1355	1.34 (0.94–1.92)	0.11	25.48	6	76	0.0003
Miscarriages	5	470	470	1.27 (0.93–1.72)	0.13	2.5	4	0	0.64
Oocytes retrieved	5	668	669	−0.04 (−1.04–0.96)	0.94	9.16	4	56	0.06
Adverse events	4	1099	1105	1.65 (1.15–2.36)	0.006	9.65	3	69	0.02

## Data Availability

The data used to support the findings of this study are included within the article.
